# Incidence of and Risk Factors for Hospital-Acquired Diarrhea in Three Tertiary Care Public Hospitals in Bangladesh

**DOI:** 10.4269/ajtmh.13-0484

**Published:** 2014-07-02

**Authors:** Mejbah Uddin Bhuiyan, Stephen P. Luby, Rashid Uz Zaman, M. Waliur Rahman, M. A. Yushuf Sharker, M. Jahangir Hossain, Choudhury H. Rasul, A. R. M. Saifuddin Ekram, Mahmudur Rahman, Katharine Sturm-Ramirez, Eduardo Azziz-Baumgartner, Emily S. Gurley

**Affiliations:** Centre for Communicable Diseases, icddr,b, Dhaka, Bangladesh; Centers for Disease Control and Prevention (CDC), Atlanta, Georgia; Khulna Medical College Hospital, Khulna, Bangladesh; Rajshahi Medical College Hospital, Rajshahi, Bangladesh; Institute of Epidemiology, Disease Control and Research, (IEDCR), Dhaka, Bangladesh

## Abstract

During April 2007–April 2010, surveillance physicians in adult and pediatric medicine wards of three tertiary public hospitals in Bangladesh identified patients who developed hospital-acquired diarrhea. We calculated incidence of hospital-acquired diarrhea. To identify risk factors, we compared these patients to randomly selected patients from the same wards who were admitted > 72 hours without having diarrhea. The incidence of hospital-acquired diarrhea was 4.8 cases per 1,000 patient-days. Children < 1 year of age were more likely to develop hospital-acquired diarrhea than older children. The risk of developing hospital-acquired diarrhea increased for each additional day of hospitalization beyond 72 hours, whereas exposure to antibiotics within 72 hours of admission decreased the risk. There were three deaths among case-patients; all were infants. Patients, particularly young children, are at risk for hospital-acquired diarrhea and associated deaths in Bangladeshi hospitals. Further research to identify the responsible organisms and transmission routes could inform prevention strategies.

## Introduction

Hospital-acquired diarrhea represents an important hazard to hospitalized patients in both high- and low-income countries, occurring in 2–32% of admitted patients in general medicine wards.^1^–^8^ In high-income countries, prolonged use of broad spectrum antibiotics, which disrupt normal colonic flora followed by colonization of *Clostridium difficile*, is the most common cause of new onset of diarrhea among hospitalized patients.^9^ Patients may face the additional risk of acquiring pathogens during contact with other patients, healthcare workers, or contaminated hospital surfaces,^10^ and in low-income hospitals, where infection control is often less rigorous,^11^ such risk could be higher. Data from high- and low-income countries suggest that age, length of hospitalization, nutritional status, immune status, and exposure to gastrointestinal procedures such as nasogastric intubation and endoscopy are host-related risk factors for hospital-acquired diarrhea.^7^,^10^,^12^

There are limited published data on incidence of hospital-acquired diarrhea in low-income countries like Bangladesh. In tertiary-level public hospitals in Bangladesh, there is ample opportunity for infection transmission through the fecal–oral route. The number of admitted patients frequently exceeds the number of available beds. Patients often share beds or are cared for on the floor. As a result of inadequate critical care units, patients with severe illnesses such as acute respiratory infection, cardiovascular disease, and stroke are often treated in the same general medicine wards where diarrheal patients are also treated.^13^ Most patients have at least one personal caregiver, usually a family member, who provides the majority of hands-on care that nursing staff would typically provide in high-income countries.^14^ Hand washing stations often lack soap and consistent water supply.^15^ Moreover, limited routine infection control in crowded wards, lead to situations where patients are at high risk of being infected by contaminated food, water, and hospital surfaces and also during contact with other patients, caregivers, and healthcare workers.^11^,^16^

Hospital-acquired diarrhea is important in Bangladeshi hospitals because new onset of diarrhea during hospitalization may cause additional medical complications for severely ill patients and pose an increased risk of mortality.^17^ Children < 5 years of age are especially susceptible to hospital-acquired infections because of a high prevalence of malnutrition^18^,^19^ and are frequently hospitalized with respiratory illnesses such as pneumonia.^20^ Previous studies in Bangladesh showed that young children with co-morbidity of diarrhea and pneumonia or diarrhea and malnutrition have an increased risk of death.^21^,^22^

Since 2007, the Institute of Epidemiology, Disease Control and Research (IEDCR) of the Government of Bangladesh and the icddr,b, has been conducting surveillance to understand the epidemiology of hospital-acquired illnesses in adult and pediatric medicine wards in three tertiary-level hospitals. Rates of hospital-acquired respiratory illness in these three tertiary-level hospitals were estimated to be six cases per 1,000 patient days^13^; in this manuscript, we report the incidence, timing, and associated risk factors for hospital-acquired diarrhea in these three Bangladeshi tertiary hospitals during April 2007 through April 2010.

## Methods

### Surveillance settings.

In April 2007, we initiated hospital-acquired illness surveillance in one adult male medicine ward and one pediatric ward in Faridpur (250 beds) and Rajshahi Medical College (500 beds) Hospitals. In May 2008, we included Khulna Medical College Hospital (250 beds) and added one adult female medicine ward in each of the three tertiary hospitals and conducted surveillance activities. Pediatric wards in Bangladesh's tertiary public hospitals typically admit patients < 14 years of age. Older patients are admitted to adult medicine wards. Though the surveillance wards consisted of 13–40 beds, on average each day 11 patients were hospitalized for every 10 beds in these wards.^13^

### Surveillance activities.

We recruited one physician among existing hospital staff to carry out surveillance activities at each ward. Each physician was provided a monthly stipend of US$45 as an incentive for data collection and was assisted by one full-time field assistant posted to each hospital (monthly salary US$335). During regular daily rounds, surveillance physicians reviewed medical records, noted clinical progression of all admitted patients to identify patients admitted for > 72 hours who developed new onset of diarrhea, defined as passage of ≥ 3 liquid stools per day.^23^ Patients who were hospitalized with diarrhea were excluded. According to the Centers for Disease Control and Prevention (CDC) guideline, hospital-acquired infections are those that occur after 48 hours of hospitalization,^24^ but we used 72 hours to increase the specificity of our definition. Because patients could develop new onset of diarrhea after hospital discharge,^25^ we defined hospital-acquired diarrhea as new onset diarrhea occurring no sooner than 72 hours after admission and no later than 48 hours after discharge. When a patient developed hospital-acquired diarrhea in the hospital, surveillance physicians recorded demographic and medical information and date of diarrhea onset.

For post-discharge follow-up, a random subset of discharged patients was identified who was hospitalized for > 72 hours, but did not develop diarrhea. Each day during June 2008 to July 2009, field assistants randomly identified one discharged patient from each study ward. Five to 7 days later, field assistants telephoned these patients to inquire if they had developed diarrhea within 48 hours of hospital discharge. Field assistants' recorded demographic and medical information, whether diarrhea developed after discharge, from each patient.

Considering the context of Bangladeshi hospital, we assumed that caregivers and visitors could transmit infection to patients during patient care. Therefore, from May 2008, we conducted a monthly cross-sectional survey among all attendants (caregivers and visitors) in each study ward and asked them if they were experiencing diarrhea on the day of the survey. This survey was conducted on the 15th of each month during peak visiting hours when the highest number of caregivers and visitors were present in the ward.

We asked healthcare workers in the study wards to report to surveillance physicians if they developed diarrhea. Additionally, we requested each surveillance physician and field assistant to follow up with these healthcare workers on a weekly basis to ask them whether they had developed diarrhea.

### Data analysis.

We used descriptive statistics to summarize demographics of hospital-acquired diarrhea case-patients. We recorded the total number of hospitalization days by each patient who stayed > 72 hours and then subtracted 3 days from hospitalization days to calculate “patient-days at risk” for each patient. We counted clusters of hospital-acquired diarrhea, defined as identification of three or more diarrhea cases among patients and/or healthcare workers in the same ward in a rolling 7-day window.

### Incidence calculation.

We calculated the crude and adjusted incidence of hospital-acquired diarrhea per 1,000 patient-days at risk using the following equation:

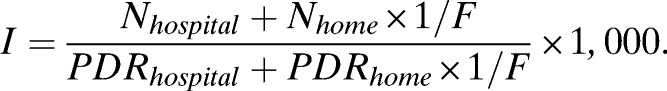

*I* = Incidence of hospital-acquired diarrhea.
*N_hospital_* = Number of diarrhea cases identified after 72 hours of hospital admission.
*N_home_* = Number of patients who developed diarrhea within 48 hours of hospital discharge.
*F* = Number of patients were followed up after discharge/(Number of patients stayed > 72 hours − *N_hospital_*).
*PDR_hospital_* = Patient-days at risk in hospital.
*PDR_home_* = Patient-days at risk after discharge of those who were followed up.

The equation provides the crude estimate of incidence of hospital-acquired diarrhea if we ignore *N_home_* and *PDR_home_*, i.e., set them to zero. Otherwise, this provides the adjusted incidence, which accounts for those patients who developed diarrhea after discharge from the hospital. We estimated *N_home_* and *PDR_home_* based on discharge data during June 2008–July 2009. We estimated 95% confidence interval (CI) for both crude and adjusted incidence assuming the incidence of case patients to have Poisson distribution. We plotted crude incidence of hospital-acquired diarrhea by month for adult and pediatric medicine wards to explore seasonal patterns. We used monthly aggregated hospital admission data to calculate bed occupancy rates for each hospital ward. We calculated the Pearson correlation coefficient between monthly bed occupancy rates and hospital-acquired diarrhea incidence each month in each ward to identify relationship between these two variables and used the *t* test to assess the statistical significance.

### Risk factors for hospital-acquired diarrhea.

We identified patients with a hospital stay of > 72 hours who did not develop diarrhea from our follow-up data set and defined them as control. We compared characteristics of hospital-acquired diarrhea patients (cases) with these controls to identify risk factors for hospital-acquired diarrhea. Because there were more adult study wards than pediatric study wards, this resulted in a higher number of controls in adult wards than in pediatric wards. We used simple and multiple logistic regression models to explore the associations between onset of hospital-acquired diarrhea and age of patient, length of hospital stay, and antibiotic exposure within 72 hours of admission. We used a *P* value cut-off point of 0.3 in simple logistic regression analysis to select covariates for multiple logistic regression.^26^ We analyzed adult and pediatric data separately during the regression analyses as we assumed that risk factors for pediatric and adult patients may be different. We accounted for ward-level clustering in the adult model because male and female wards were independent wards.

### Ethical considerations.

We obtained informed written consent from patients or their parents (if participants were minors) including the participants who were selected for a follow-up call after hospital discharge. Our surveillance protocol was approved by the institutional review board of icddr,b.

## Results

During April 2007 through April 2010, 97,932 patients were admitted to our surveillance wards, and the 23,004 (23%) hospitalized for > 72 hours contributed 80,398 patient-days at risk. Of 23,004 patients, 257 (1%) developed new onset of diarrhea during their hospital stay (Table 1). The median age of hospital-acquired diarrhea case-patients was 8 months (interquartile range [IQR]: 4–24 months) among children and 40 years (IQR: 28–60 years) among adults. Fifty-three (40%) of 131 pediatric case-patients were admitted with respiratory illnesses including pneumonia, bronchial asthma, and other respiratory conditions (Table 1). Of 257 case-patients, 207 (81%) received antibiotics within 72 hours of hospitalization, 239 (93%) were discharged, and 107 (45%) of 239 were discharged within 2 days after developing diarrhea. There were three deaths among case-patients (1%); all were infants: one 6 months of age and the other two 7 months of age. The deaths occurred in January 2009, December 2009, and January 2010. Of the three deaths, one was admitted for pneumonia, another for protein-energy malnutrition, and the third for meningitis.

The crude incidence of hospital-acquired diarrhea was 3.2 cases per 1,000 patient-days at risk (Table 2). During the 14-month follow-up period, field assistants contacted 2,367 discharged patients and identified 30 (1.3% [95% CI: 0.8–1.8%]) patients developed diarrhea within 48 hours after discharge. We estimated 601(95% CI: 494–709) hospital-acquired diarrheal cases (Table 2), which represented 2.6% (95% CI 2.4–2.8%) of all patients hospitalized for > 72 hours within the three hospitals. The overall adjusted incidence of hospital-acquired diarrhea was 4.8 cases per 1,000 patient-days at risk (95% CI: 4.0–5.7) (Table 2).

**Figure 1.**
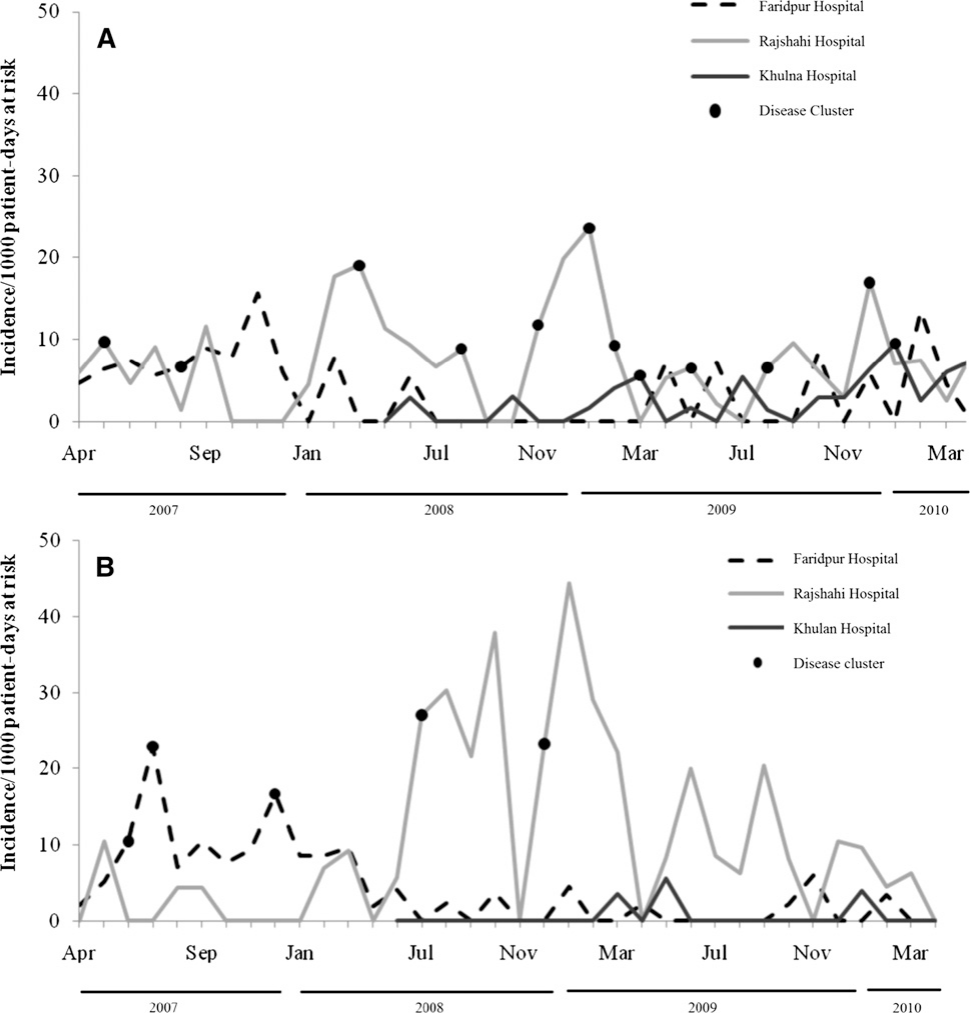
(**A**) Crude incidence of hospital-acquired diarrhea per 1,000 patient-days at risk among hospitalized pediatric patients in Bangladesh, by month and hospital, 2007–2010. (**B**) Crude incidence of hospital-acquired diarrhea per 1,000 patient-days at risk among hospitalized patients in male medicine wards (we did not plot incidence data by month in adult female medicine wards because cases occurred infrequently) in Bangladesh, by month and hospital, 2007–2010.

The crude incidence of hospital-acquired diarrhea varied between months, hospitals, and wards (Figure 1A and B). The highest incidence of hospital-acquired diarrhea in pediatric wards (Figure 1A) and in adult male medicine wards (Figure 1B) occurred in January 2009. During these two highest incidence months, 8% of pediatric patients (8 of 102) and 8% (2 of 25) of adult male patients who stayed > 72 hours in the surveillance wards developed hospital-acquired diarrhea. We found no correlation between monthly bed occupancy rate and incidence of hospital-acquired diarrhea in our hospital wards (Pearson correlation coefficient, r = 0.28, *P* value = 0.09).

We identified 10 (4%) diarrhea cases among 269 healthcare staff working in the study wards, including seven physicians, one nurse, and two support staff. Eight healthcare workers were from the pediatric wards and two were from the adult medicine wards. Healthcare staff worked for a mean of 2.5 days (range: 2–3) while having diarrheal illness.

A total of 19 hospital-acquired diarrhea clusters were identified; 13 (64%) were from pediatric wards (Table 2). Sixty-four (25%) of 257 case-patients and five healthcare workers were part of the disease clusters. Three clusters contained both case-patients and healthcare workers. The mean number of case-patients in each cluster was 4 (range: 3–6). More than 50% (7 of 13) of disease clusters in pediatric wards occurred during November–February (Figure 1A). Two of the three case-patients' deaths were occurred among these clusters.

During May 2008 through April 2010, we conducted 72 monthly cross-sectional surveys on attendants in pediatric wards and 138 similar surveys in adult medicine wards. At the time of each survey, there was an average of 1.67 attendants or visitors for each patient in the ward. A higher proportion of attendants in pediatric wards (60 of 4,181, 1.4%) reported having diarrhea on the day of the survey than attendants in adult wards (52 of 8,754, 0.6%) (*P* < 0.001). A higher proportion of surveys in pediatric wards (38 of 72, 53%) had at least one attendant who reported having diarrhea compared with adult ward surveys (37 of 138, 27%) (*P* < 0.001).

Children < 1 year of age were more likely to develop hospital-acquired diarrhea than children 5–13 years of age (odds ratio [OR] = 6.6; 95% CI: 3.8–11.4) (Table 3). Among children, the risk of developing hospital-acquired diarrhea increased with each additional day stay in hospital beyond 72 hours (OR = 1.13; 95% CI: 1.09–1.18). Children who received antibiotics within 72 hours of admission were less likely to develop diarrhea than to children who did not receive antibiotics (OR = 0.3; 95% CI: 0.2–0.6) (Table 3). Among adults, the risk of developing hospital-acquired diarrhea increased with each additional day stay in hospital beyond 72 hours (OR = 1.12; 95% CI: 1.04–1.20). Adults who received antibiotics within 72 hours of admission were less likely to develop diarrhea than patients who did not receive antibiotics (OR = 0.7; 95% CI: 0.6–0.9) (Table 4).

## Discussion

In our study, 26 of every 1,000 patients hospitalized for > 72 hours developed hospital-acquired diarrhea. Infants (children < 1 year of age) were at highest risk for hospital-acquired diarrhea during their hospitalization, a finding consistent with previous studies^5^,^12^,^27^,^28^; all three deaths in this study occurred among infants, suggesting that new onset of diarrhea during hospitalization could lead to particularly poor health outcomes in very young children. The proportion of patients who developed hospital-acquired diarrhea in this study was similar to other studies from middle- and low-income countries.^2^,^3^,^8^ These data can help prioritize infection control initiatives in Bangladeshi hospitals and provide baseline rates of infection that could be used to measure effectiveness of infection control interventions. However, given that conducting this surveillance activity required US$470 per month per hospital of external funding for the data collection staff alone, this surveillance model is unlikely sustainable in this or other resource-poor settings. Nonetheless, these surveillance methods are still less costly than other laboratory-based surveillance models and could be used to generate baseline rates in similar settings where no data currently exist.

In these study hospitals, the risk of developing diarrhea during hospital stay increased with each additional day of hospitalization after > 72 hours of stay. Longer stays in hospital wards with limited routine infection control may increase the possibility of exposure to potential pathogens at hospital settings,^7^,^29^ which might cause new onset of diarrhea. Additionally, we found that many caregivers had diarrhea while caring for ill patients, which also pose risk to patients.

Overcrowding in hospital wards has been identified as an important factor associated with increased burden of healthcare-associated infections in developing countries^30^; in this study, we did not find any correlation between bed occupancy and incidence of hospital-acquired diarrhea in our study wards. The bed occupancy rates in these hospital wards are consistently > 100% and the presence of visitors and attendants further increased the density of people on the wards. Crowding may contribute to an increased risk in our wards compared with other settings, however the relative differences month-to-month in our ever-crowded wards were not associated with increased risk.

In our study population, patients who used antibiotics were less likely to develop hospital acquired diarrhea. The protective association between antibiotic exposure and hospital-acquired diarrhea identified in our study, to our knowledge, has not been previously reported in low-income settings. In middle- and high-income countries, antibiotic exposure increases risk of hospital-acquired diarrhea.^31^ Use of broad spectrum antibiotics favors growth of *C. difficile* that can cause diarrhea during hospitalization.^9^ The observed protective role of antibiotics in this study suggest that *C. difficile* may not be an important cause of diarrhea in these hospitals and further supports the role of other enteric pathogens^29^ in causing hospital-acquired diarrhea in these hospitals. One explanation for this unexpected finding could be that the relationship between antibiotic use among patients and acquisition of diarrheal illness is confounded by poverty. Patients in Bangladesh typically have to buy their own medicines,^32^ and receiving no antibiotics could be a marker of poverty, which may be more common among malnourished children who are at increased risk of hospital-acquired infection. It is also possible that antibiotic exposure reduces the risk of diarrhea similar to the protection that prophylactic antibiotics provide travelers to low-income countries. Indeed, antibiotic prophylaxis among travelers is associated with a 65–90% reduction in diarrhea^33^,^34^; we are not recommending prophylactic use of antibiotics based on our study findings; however, clinical trials to understand the potential efficacy of antibiotics to reduce hospital-acquired diarrhea in these health facilities would be informative.

In Bangladesh, 41% of children < 5 years of age suffer from malnutrition and the prevalence is highest among children < 24 months of age.^18^ Malnourished children are at increased risk for hospital-acquired infections including hospital-acquired gastroenteritis compare with well-nourished children.^12^,^19^,^35^ Our study did not systematically collect data on malnutrition of patients therefore we are unable to investigate the association between malnutrition and hospital-acquired diarrhea. We were also unable to confirm the role of hospital-acquired diarrhea in the death of one infant hospitalized for malnutrition, but malnourished children are at increased risk of mortality from community-acquired diarrheal illness in Bangladesh^22^,^36^,^37^. We also identified one death in an infant hospitalized with pneumonia. A study in Bangladesh suggested that concurrent diarrhea posed an increased risk of complication and mortality among young children with pneumonia.^21^ Recording cause of death of children who develop hospital-acquired diarrhea would be useful in the future.

In the pediatric wards, we identified hospital-acquired diarrhea cases year-round, yet cases increased during Bangladesh's winter months of November through February. In addition, more than half of the hospital-acquired diarrhea clusters and all deaths among case-patients occurred during those months. These findings suggest that children in Bangladesh may be at increased risk for hospital-acquired diarrhea and associated death during winter. Unlike pediatric wards, we did not identify a seasonal pattern of hospital-acquired diarrhea illness in adult medicine wards. We found that adult female medicine wards at each hospital had the lowest rate of hospital-acquired diarrhea, suggesting that adult females had a lower risk for hospital-acquired diarrhea than other patients. We found no difference in the infrastructure, facilities, or crowding in the adult female medicine wards compared with other study wards that could explain the lower incidence among adult female patients. The proportion of patients admitted with community-acquired diarrhea on the ward could also play a role in transmission; we have not measured this on any of our study wards, however do not have any reason to believe that this proportion would be lower in female medicine wards compared with others. The other possibility is that these findings are the result of under-reporting in female wards; although the mechanism of under-reporting is unclear, female patients may be less likely to report new symptoms to predominantly male physicians. Future surveillance activities should investigate why risk may be lower in female wards, including possible under-reporting.

During the study period, healthcare workers reported diarrheal illness and were also associated with hospital-acquired diarrhea clusters. Healthcare staff with diarrhea often continued to provide care during their illness, thereby increasing the possibility of spreading infection to others.

Several limitations of this study should be noted. We defined hospital-acquired diarrhea as new onset of diarrhea from 72 hours post-admission through 48 hours post-discharge. The incubation period of gastrointestinal pathogens ranges from 8 hours to 14 days^38^; therefore, we likely missed some patients who developed new onset of diarrhea in the first 72 hours of admission or after 2 days post-hospital discharge and may have also included infections that were incubating before hospitalization. Our definition, however, covered the incubation period of common diarrheal pathogens found in Bangladesh,^39^,^40^ including *Salmonella typhi*, *Shigella* sp., *Campylobacter* sp., enterohemorrhagic *E. coli* (EHEC), and rotavirus,^38^,^41^ suggesting that cases we missed or patients we falsely categorized as cases likely had little impact on our estimation. We identified cases during post-discharge follow-up, which indicates that illness occurs post-discharge and including these additional cases provides a better estimate of hospital-acquired diarrheal rates than crude rates. However, a second limitation of the study was that we collected post-discharge data for only 1 year and adjusted our incidence based on single year of assessments. Although we followed a large number of patients in that year, the incidence rate changed over the study period (Figure 1A and B) indicating that the risk during the year with follow-up activities might not have been representative of the risk during the entire study period. We also lacked laboratory data, which prevented describing pathogens associated with hospital-acquired diarrhea, and confirming that clusters of illnesses were related. Laboratory-based surveillance requires more resources than are typically available in low-income countries. Nevertheless, identifying a low-cost method to detect pathogens associated with diarrheal outbreaks would be useful to further characterize the epidemiology of hospital-associated diarrhea in these settings and better develop infection control policies.

This study shows that hospitalized patients, specifically young children, are at risk for hospital-acquired diarrhea in resource-poor hospital settings. Infants were at highest risk for hospital-acquired diarrhea and associated death. Further research to identify the responsible organisms and transmission routes could inform prevention strategies.

## Figures and Tables

**Table 1:** Characteristics of hospital-acquired diarrhea cases identified at three tertiary-level hospitals in Bangladesh, 2007–2010

Characteristics	Value	
	Pediatric wards (*N* = 131)	Adult wards (*N* = 126)
Days from hospital admission to new onset of diarrhea, median (IQR)	5 (4–6)	5 (4–6)
Hospital outcomes		
Discharged	118 (90)	121 (96)
Left against medical advice	8 (6)	5 (4)
Transferred to other facility/ward	2 (2)	0
Death	3 (2)	0
Days from new onset of diarrhea to hospital outcome, median (IQR)	2 (2–5)	2 (2–5)
Antibiotics* received within 72 hours of admission	110 (84)	97 (77)
Admitting diagnosis		
Pneumonia, bronchial asthma, and other respiratory diseases	53 (40)	26 (21)
Encephalitis, meningitis, and other nervous system diseases	21 (16)	13 (10)
Nephrotic syndrome, nephritis, and other renal diseases	17 (13)	2 (2)
Birth asphyxia, pre-term condition, and other neonatal illnesses	11 (8)	0 (0)
Anemia, leukemia, and other hemolytic diseases	9 (7)	9 (7)
Malnutrition	7 (5)	0 (0)
Malaria, dengue, typhoid, and other febrile diseases	5 (4)	13 (10)
Gastro-intestinal diseases (excluding diarrhea)	1 (1)	22 (17)
Myocardial infarction, stroke, and other cardiovascular diseases	0 (0)	23 (18)
Poisoning	2 (2)	2 (2)
Animal/insect bite	1 (1)	0 (0)
Diabetes	0 (0)	1 (1)
Arthritis	0 (0)	2 (2)
Other conditions	3 (2)	10 (8)
Missing diagnosis	1 (1)	3 (2)

Note: Data are no. (%) of patients, unless otherwise indicated.

* The prevalent classes of antibiotics prescribed were macrolides, cephalosporins, penicillins, tetracyclines, and quinolones.

IQR = interquartile range.

**Table 2:** Incidence of hospital-acquired diarrhea and frequency of disease clusters in three tertiary-level hospitals in Bangladesh, by hospital ward, 2007–2010*

Characteristic	Faridpur Hospital			Rajshahi Hospital			Khulna Hospital			Total
	Adult males	Adult females	Pediatric	Adult males	Adult females	Pediatric	Adult males	Adult females	Pediatric	
Number of patients admitted > 72 hours	4,443	1,811	1,799	2,410	1,077	5,128	2,078	1,648	2,610	23,004
Mean bed occupancy rate	182	238	132	109	152	157	141	144	100	151
Hospital-acquired diarrhea cases identified at hospital, *N* (%)	51 (1.1)	10 (0.6)	19 (1.1)	52 (2.1)	4 (0.4)	81 (1.6)	4 (0.2)	5 (0.3)	31 (1.2)	257 (1.1)
Percentage of patients who developed diarrhea within 48 hours of hospital discharge, 95% CI	2.7, 1–5.6	1.5, 0.3–4.2	1.1, 0.1–4.1	0.7, 0–2.3	0, 0–0	1.4, 0.4–3.1	2.4, 0.8–5.2	1.0, 0.1–3.5	1.0, 0.2–3.0	1.3, 0.8–1.8
Number of estimated hospital-acquired diarrhea cases after adjustment, 95% CI	171, 81–261	36, 6–67	39, 10–69	67, 42–93	4, 0–8	150, 87–212	54, 14–95	21, 7–63	58, 26–90	601, 494–709
Crude incidence,	3.7,	1.7,	3.8,	7.3,	1.0,	4.6,	0.5,	0.7,	2.8,	3.2,
95% CI	2.7–4.8	0.8–3.2	2.3–6.0	5.6–9.6	0.3–2.6	3.6–5.7	0.1–1.3	0.2–1.7	1.9–4.0	2.8–3.6
Adjusted incidence,	7.6,	3.9,	4.6,	5.7,	0.7,	5.4,	4.4,	2.1,	3.6,	4.8,
95% CI	3.6–11.6	0.6–7.1	1.2–8.1	3.5–7.9	0–1.3	3.1–7.6	1.1–7.7	0.7–6.1	1.6–5.5	4.0–5.7
Number of clusters	3	0	1	2	1	10	0	0	2	19

* CI = confidence interval.

**Table 3:** Factors associated with hospital-acquired diarrhea among pediatric patients in three tertiary-level hospitals in Bangladesh, 2007–2010

Exposure	Cases (*N* = 141)	Controls (*N* = 810)	Simple logistic regression		Multiple logistic regression	
	n (%)	n (%)	OR	95% CI	OR	95% CI
Age categories, years						
< 1	83 (59)	273 (32)	4.3	2.6–7.1*	6.6	3.8–11.4*
1–4	36 (26)	216 (27)	2.4	1.4–4.1†	2.9	1.6–5.3*
5–13	22 (16)	316 (39)	Reference		Reference	

Days of hospitalization after > 72 hours	141	810	1.10	1.06–1.14*	1.13	1.09–1.18*

Antibiotic received within 72 hours of hospital admission						
No	22 (16)	72 (9)	Reference		Reference	
Yes	119 (84)	738 (91)	0.5	0.3–0.9†	0.3	0.2–0.6*

* *P* < 0.001;

† *P* < 0.05.

**Table 4:** Factors associated with developing hospital-acquired diarrhea among adult medicine ward patients in three tertiary-level hospitals in Bangladesh, 2007–2010

Exposure	Cases (*N* = 146)	Controls (*N* = 1503)	Simple logistic regression*		Multiple logistic regression*	
	n (%)	n (%)	OR	95% CI	OR	95% CI
Age categories, years						
14–30	46 (32)	501 (34)	Reference		Reference	
31–45	34 (23)	397 (27)	0.9	0.8–1.0	0.9	0.6–1.2
46–65	46 (32)	429 (29)	1.1	0.6–2.4	1.1	0.4–2.9
> 65	19 (13)	151 (10)	1.3	1.1–1.6	1.3	0.9–1.9

Days of hospitalization after > 72 hours	146	1503	1.12	1.04–1.20	1.12	1.04–1.20

Antibiotic received within 72 hours of hospital admission						
No	29 (20)	222 (15)	Reference		Reference	
Yes	117 (80)	1281 (85)	0.7	0.4–1.0	0.7	0.6–0.9

* Ward-level clustering was considered.

† *P* < 0.05.
